# Supplementary material for: Comparative multiomics analysis of cell physiological state after culture in a basket bioreactor
Source: Sci Rep. 2022 Nov 23;12:20161. doi: 10.1038/s41598-022-24687-4 (PMC9686226; doi:10.1038/s41598-022-24687-4)

66041.1:163125051:163125095:-@NW_023666041.1:163124719:163124746:-@NW_023666041.1:163123209:163

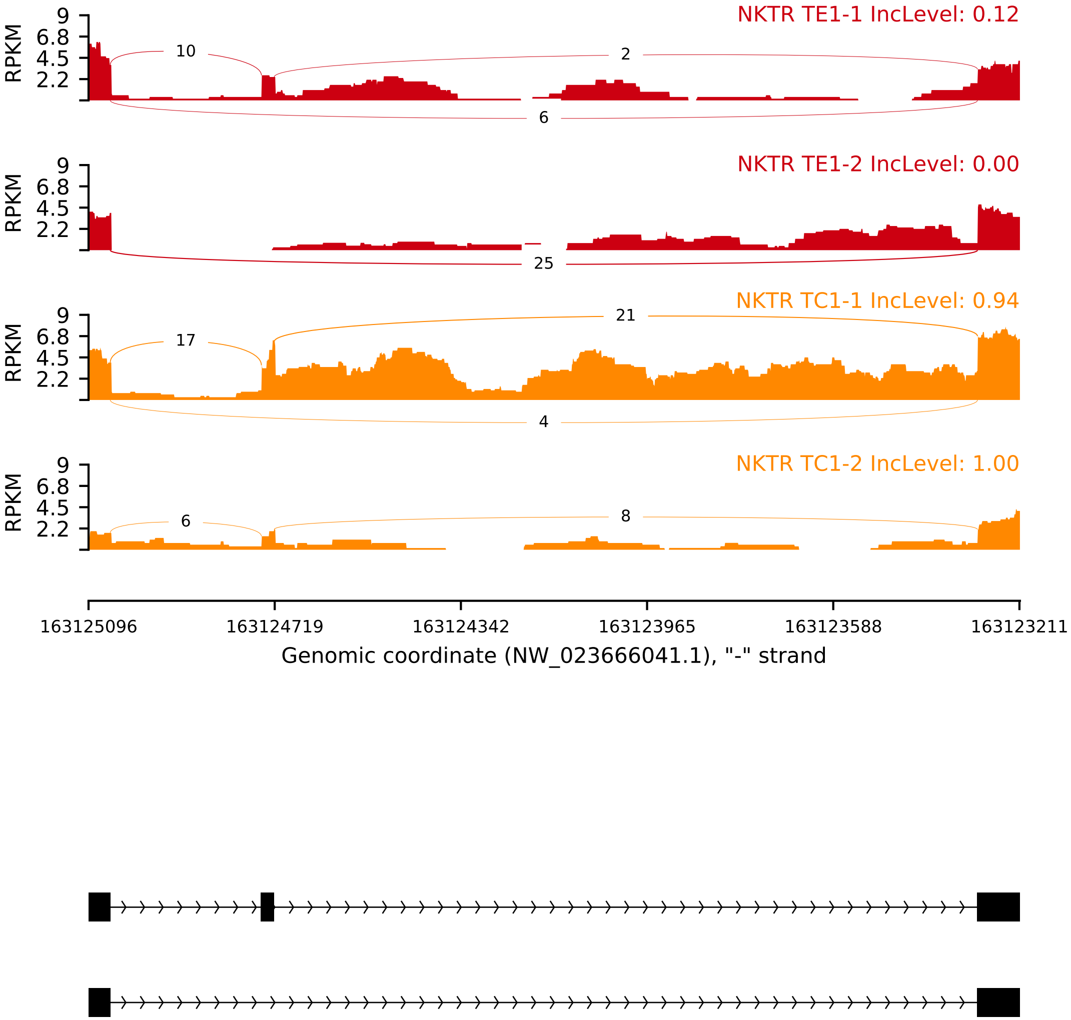

Supplement: Supplementary file 1 — Supplementary Information 1. [file 41598_2022_24687_MOESM1_ESM.zip › raw data/Transcriptomic raw data/7.AS/2.dasplot/TE1vsTC1/SE/7_NKTR_NW_023666041.1_163125051_163125095_-@NW_023666041.1_163124719_163124746_-@NW_023666041.1_163123209_163123296_-.pdf]
